# Chemical pancreatectomy in non-human primates ablates the acini and ducts and enhances beta-cell function

**DOI:** 10.1038/s41598-023-35820-2

**Published:** 2023-06-05

**Authors:** Ranjeet S. Kalsi, Alexander M. Kreger, Mohamed Saleh, Shiho Yoshida, Kartikeya Sharma, Joseph Fusco, Jami L. Saloman, Ting Zhang, Madison Thomas, Anuradha Sehrawat, Yan Wang, Jason Reif, Juliana Mills, Sarah Raad, Bugra Zengin, Ana Gomez, Aatur Singhi, Sameh Tadros, Adam Slivka, Farzad Esni, Krishna Prasadan, George Gittes

**Affiliations:** 1 Division of Pediatric Surgery, UPMC Children’s Hospital of Pittsburgh, 4401 Penn Avenue, Pittsburgh, PA 15224 USA; 2 Division of Pediatric Endocrinology, UPMC Children’s Hospital of Pittsburgh, Pittsburgh, PA USA; 3 Department of Medicine, Division of Gastroenterology, Hepatology, and Nutrition, Department of Neurobiology, Pittsburgh Center for Pain Research, University of Pittsburgh School of Medicine, Pittsburgh, PA USA; 4 Division of Pediatric Pathology, UPMC Children’s Hospital of Pittsburgh, Pittsburgh, PA USA; 5 Divisions of Anatomic Pathology and Molecular Genomic Pathology, University of Pittsburgh Medical Center, Pittsburgh, PA USA; 6 Department of Pediatric Radiology, UPMC Children’s Hospital of Pittsburgh, Pittsburgh, PA USA; 7 Division of Gastroenterology, Hepatology and Nutrition, University of Pittsburgh Medical Center, Pittsburgh, PA USA

**Keywords:** Islets of Langerhans, Pancreas, Pancreatitis, Oesophagogastroscopy, Pancreas, 3-D reconstruction, Endoscopy, X-ray tomography

## Abstract

Chronic pancreatitis is a debilitating disease affecting millions worldwide. These patients suffer from bouts of severe pain that are minimally relieved by pain medications and may necessitate major surgeries with high morbidity and mortality. Previously, we demonstrated that “chemical pancreatectomy,” a pancreatic intraductal infusion of dilute acetic acid solution, ablated the exocrine pancreas while preserving the endocrine pancreas. Notably, chemical pancreatectomy resolved chronic inflammation, alleviated allodynia in the cerulein pancreatitis model, and improved glucose homeostasis. Herein, we extensively tested the feasibility of a chemical pancreatectomy in NHPs and validated our previously published pilot study. We did serial computed tomography (CT) scans of the abdomen and pelvis, analyzed dorsal root ganglia, measured serum enzymes, and performed histological and ultrastructural assessments and pancreatic endocrine function assays. Based on serial CT scans, chemical pancreatectomy led to the loss of pancreatic volume. Immunohistochemistry and transmission electron microscopy demonstrated exocrine pancreatic ablation with endocrine islet preservation. Importantly, chemical pancreatectomy did not increase pro-nociceptive markers in harvested dorsal root ganglia. Also, chemical pancreatectomy improved insulin secretion to supranormal levels in vivo and in vitro. Thus, this study may provide a foundation for translating this procedure to patients with chronic pancreatitis or other conditions requiring a pancreatectomy.

## Introduction

Chronic pancreatitis affects over 250,000 people in the United States and millions worldwide^1–3^. The global incidence of chronic pancreatitis is ten cases per 100,000 individuals of the general population annually, with a prevalence of 42 per 100,000 persons. Longstanding chronic pancreatitis leads to debilitating chronic pain, exocrine pancreatic insufficiency, and a brittle form of diabetes^4^. These long-term sequelae of chronic pancreatitis are challenging to treat and expensive to manage over a patient’s disease progression. Chronic pain often requires treatment with opioids, frequently leading to dependence and addiction^5,6^. In addition, patients often have exocrine pancreatic insufficiency necessitating lifelong pancreatic enzyme supplementation. Pancreatogenic diabetes occurs in approximately 80% of patients with chronic pancreatitis, and it is difficult to treat^7^. The inflammatory environment of chronic pancreatitis is thought to lead to the loss of both insulin-producing beta-cells and glucagon-producing alpha-cells. Typically, glucagon reverses hypoglycemia; however, in patients with pancreatogenic diabetes, a small insulin overdose resulting in hypoglycemia may be life-threatening^8^. Other severe complications of chronic pancreatitis include pseudocyst formation, pancreatic ascites, metabolic bone disease, splenic vein thrombosis, and biliary obstruction^9^. Furthermore, patients with chronic pancreatitis have approximately an eightfold increased risk of developing pancreatic ductal adenocarcinoma^10–12^.

In addition to managing the sequelae of chronic pancreatitis, more invasive options for treatment are often necessary, including major surgeries such as partial or total pancreatectomy that ultimately lead to a loss of islets and altered endocrine function^13^. Total pancreatectomy with islet auto-transplantation (TPIAT) has emerged as a treatment that addresses chronic pancreatitis-associated pain while attempting to preserve endocrine function, but it remains a procedure with high morbidity, substantial islet loss during islet isolation, and a 5% 30-day mortality^14–16^. Unfortunately, many continue to have pain after surgery. Approximately 20–40% of adults continue to require analgesics, and 59–70% of these patients are insulin dependent after surgery^17^.

Our lab recently developed a novel therapeutic option to treat chronic pancreatitis by using an infusion of acetic acid (AcA) into the pancreatic duct. In mice, this procedure led to near-complete ablation of the exocrine pancreas while preserving the islets^18^. This technique, termed a “chemical pancreatectomy,” also relieved chronic pancreatitis-associated pain behaviors in mice. Remarkably, it improved glucose tolerance and insulin secretion in vivo to supranormal levels. Chronic pancreatitis-associated diabetes is thought to occur due to the surrounding inflammation. Thus, removing the offending exocrine pancreas may protect islets from further damage, preserving and even improving existing endocrine function.

To translate this therapeutic approach to one day treat chronic pancreatitis in humans, we analyzed the histological, anatomical, and islet functional changes accompanying chemical pancreatectomy in normal non-human primates (NHPs). Anatomically, the primate pancreas is similar to the human pancreas. In humans, the pancreatic duct is routinely accessed via a non-surgical endoscopic technique called endoscopic retrograde cholangiopancreatography (ERCP). However, given the small size of the primates, our attempts at ERCP were unsuccessful in selectively cannulating the pancreatic duct, which necessitated a laparotomy similar to that required in mice. In NHPs, the chemical pancreatectomy demonstrated excellent ablation of the exocrine pancreas. There was neither overt pain nor nerve injury, and islets were preserved, exhibiting improved (supranormal) glucose homeostasis and insulin secretion like the mice.

## Results

### Gross evaluation and imaging of the pancreas in NHPs after chemical pancreatectomy

**Figure 1:**
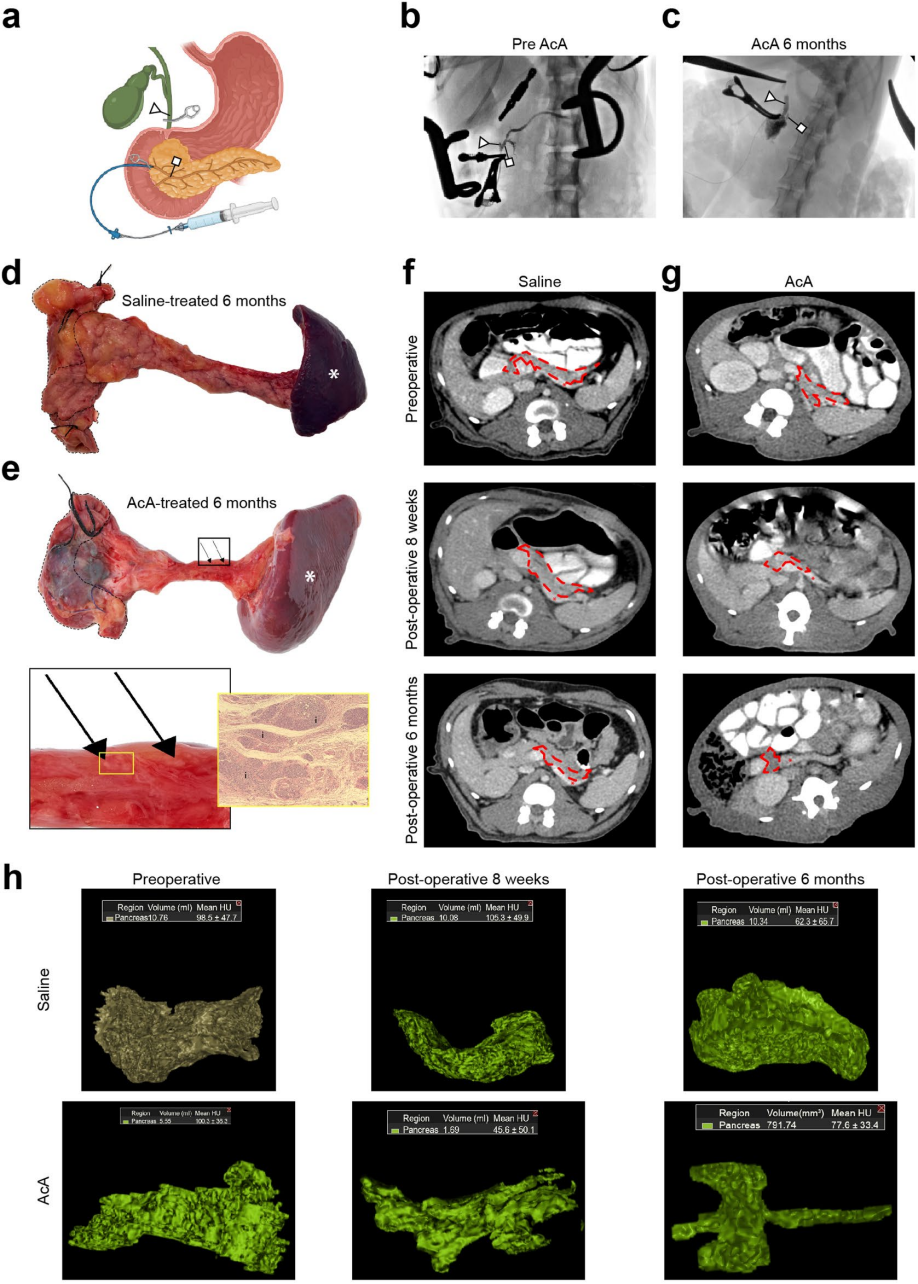

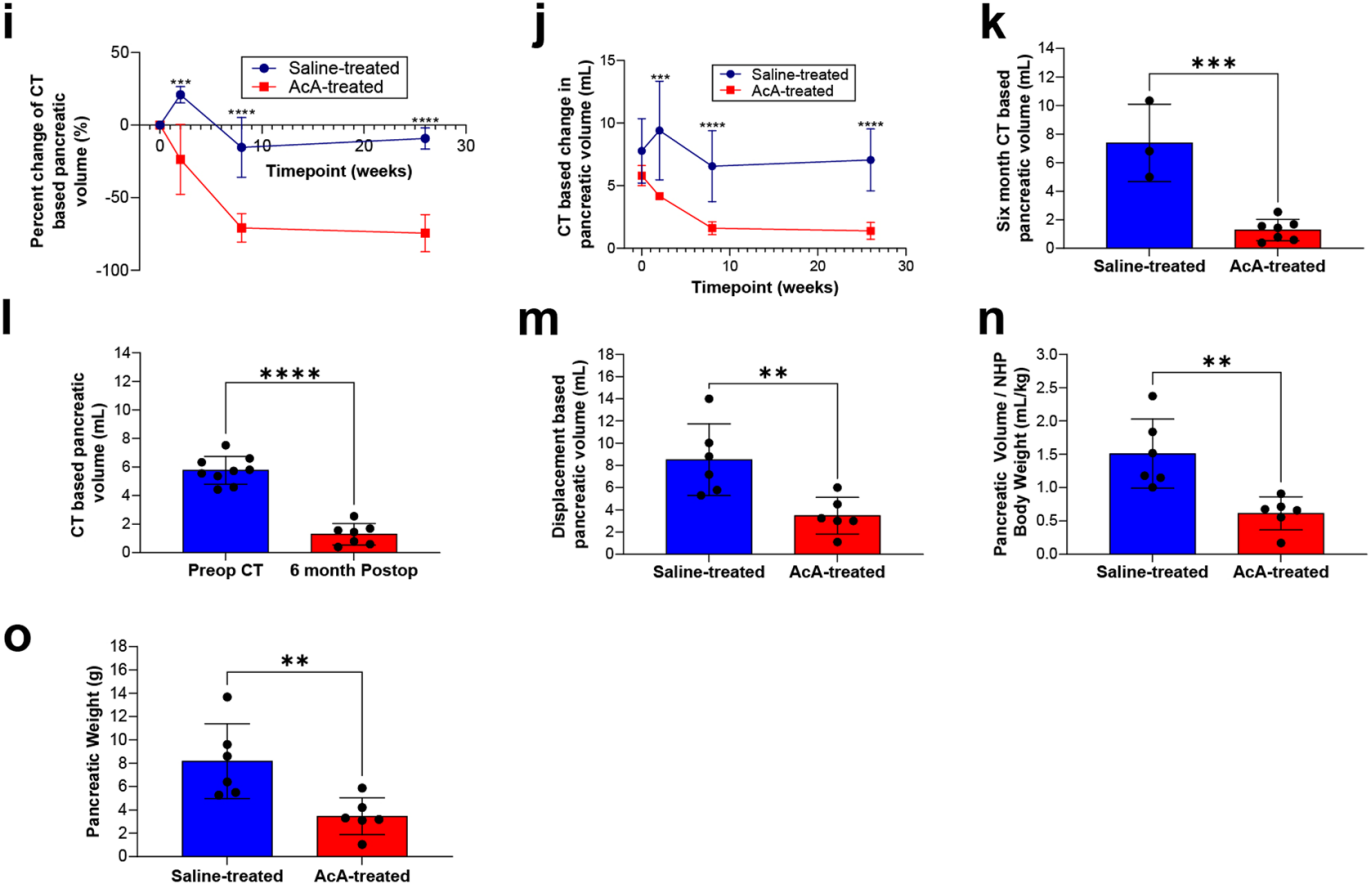
Gross evaluation and imaging of the pancreas in NHPs after chemical pancreatectomy. (**a**) Graphic illustration demonstrates placement of clamps and the typical set-up prior to infusion. (**b**) Pancreatography prior to AcA infusion, depicting a patent CBD and pancreatic duct. (**c**) Pancreatography of the same NHP six months after AcA infusion demonstrated normal filling of the CBD and proximal pancreatic duct and no filling of the distal pancreatic duct. (a, b, and c) The triangle arrowhead denotes CBD. The square arrowhead indicates the pancreatic duct. (**d**) Gross morphology of a NS-infused pancreas six months after surgery. (**e**) Gross morphology of an AcA-infused pancreas six months after surgery with a magnified view. The exocrine pancreas is nearly replaced by fatty tissue with apparent clusters of islets (arrows) and corresponding histology showing islets (i). (**d** and **e**) A dashed outline denotes the duodenum with suture ligating the proximal and distal ends of the lumen. The normal spleen remains attached (asterisk). (**f** and **g**) CT scan demonstrates changes of the pancreas after NS (**f**) or AcA (**g**) infusion over time, respectively. The dashed outline indicates the pancreas, while the asterisk denotes the splenic vein. (**h**) Representative samples of reconstructed 3D images of saline-treated and AcA-treated pancreata. (**i** and **j**) CT-based percentage change in pancreatic volume over time and CT-based pancreatic volume over time comparing saline-treated to AcA-treated NHPs (Repeated Measure ANOVA with Šídák's multiple comparisons test ***P = .0006, ****P < .0001, saline-treated n = 3, AcA-treated n = 9). (**k**) CT-based pancreatic volume after six months comparing saline-treated to AcA-treated NHPs (unpaired t-test ***P = .0004). (**l**) CT-based pancreatic volume comparing six months after AcA to preoperative baseline (paired t-test ****P < .0001). (**m**) Evaluation of pancreatic volume by displacement after harvest (unpaired t-test **P = .0067). (**n**) Assessment of pancreatic volume (by displacement) corrected for NHP body weight (unpaired t-test **P = .0034). (**o**) Evaluation of pancreatic weight at the time of sacrifice was significantly decreased in the AcA-treated NHPs compared to the saline-treated NHPs (unpaired t-test **P = .0088).

We have previously demonstrated successful ablation of the exocrine pancreas with preservation of the endocrine pancreas in mice with chronic pancreatitis. Herein, we further showed similar success in NHPs. The NHPs underwent intrapancreatic ductal infusion of normal saline (NS) or 2% AcA (Fig. 1a). Iodine contrast infusion pancreatography ensured correct positioning of the catheter, allowing for infusion down the pancreatic duct without duodenal spillage or flow beyond our bulldog clamp on the common bile duct (Fig. 1b, Supplementary Fig. 1A,B, Supplemental Video 1). Six months after the chemical pancreatectomy, pancreatography showed proximal filling of a short segment of the pancreatic duct with the remainder of the duct not visualized (Fig. 1c), indicating ductal obliteration. Six months after surgery, AcA-treated pancreata were atrophied compared to saline-infused pancreata, which appeared normal in size and looked pink to tan (Fig. 1d). The AcA-treated pancreas appeared as a scant membrane that laid as a thin veil over the splenic vein with grossly apparent clusters of islets, which were histologically confirmed (Fig. 1e).

A baseline CT of the NHP abdomen and pelvis was obtained before surgery to identify pancreatic or intraabdominal abnormalities and to calculate the pancreatic volume. We generated 3D reconstructions of the pancreas from the CT scans before surgery and at two weeks, eight weeks, and six months after surgery (Fig. 1f–h, Supplemental Videos 2–4). In the saline-treated NHPs, the pancreatic volume slightly increased two weeks after the infusion, which is consistent with edema of the pancreas following infusion (Fig. 1i,j, Supplementary Fig. 2A). In the AcA-treated NHPs, pancreatic volume was reduced at two weeks. The CT-based volumetric analysis showed a significant loss of pancreatic volume in the AcA-treated pancreata compared to controls beginning eight weeks after the infusion. This volume loss persisted six months after the infusion (Fig. 1i–k, Supplementary Fig. 2b). Compared to baseline, there was a 70% reduction in the volume of the AcA-treated pancreas at eight weeks and six months (Fig. 1l and Supplementary Fig. 2C). At the six-month time point, the saline-treated and AcA-treated NHPs had comparable body weights (Supplementary Fig. 2D). However, pancreatic volume assessed by volumetric displacement and pancreatic volume standardized to body weight revealed a statistically significant decrease in pancreatic volume in AcA-treated NHPs compared to saline-treated NHPs (Fig. 1m,n). Similarly, pancreatic weight after chemical pancreatectomy was significantly lower than the saline-treated group (Fig. 1o). Also, the CT scan-based pancreatic volume calculations were comparable to the actual volumes of the harvested pancreata as measured by volumetric displacement at pancreatic harvest (Supplementary Fig. 2E). A table with individual NHP body weights and pancreatic measurements is included separately (Supplementary Table 1).

### Histological changes of NHP pancreas six months after pancreatic intraductal infusion

**Figure 2:**
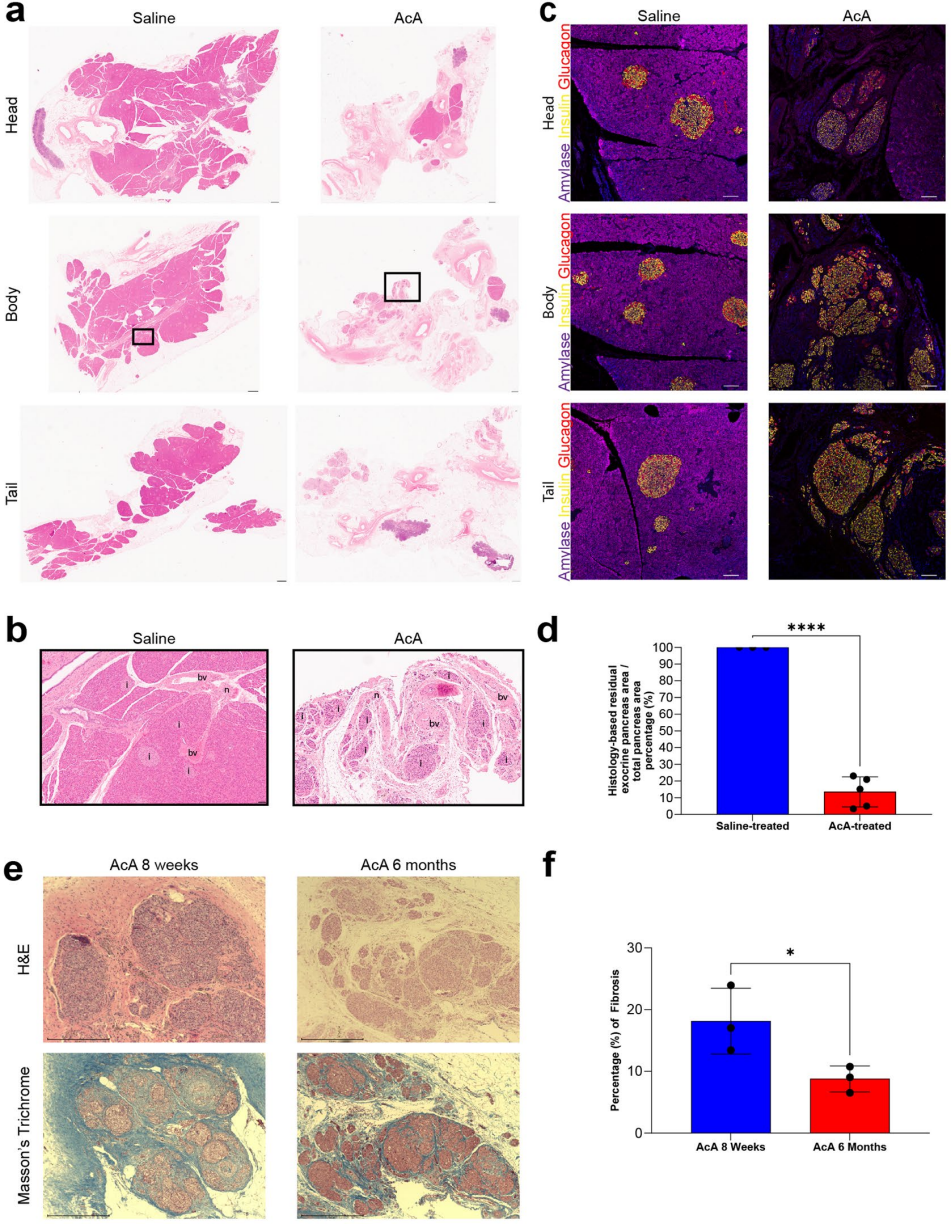
Histological and immunohistochemical evaluation of the pancreas after chemical pancreatectomy. (**a**) Panoramic H&E view of saline-treated and AcA-treated NHP of the pancreatic head, body, and tail. Scale bar: 10 mm. (**b**) Magnified views of saline-treated and AcA-treated NHP pancreatic bodies demonstrate normal islets (i), blood vessels (bv), and nerves (n). Scale bar: 400 µm. (**c**) Saline-treated NHP pancreas demonstrates normal endocrine and exocrine pancreas histology. NHPs after chemical pancreatectomy demonstrate a small amount of amylase in the head of the pancreas, but there is no amylase in the body or tail. Normal insulin and glucagon immunostaining is seen throughout the pancreas. Scale bar: 100 µm (**d**) Quantification and comparison of exocrine pancreas based on histology (unpaired t-test ****P < .0001). (**e**) H&E and Masson’s Trichrome staining demonstrate significant fibrosis eight weeks after chemical pancreatectomy. By six months after chemical pancreatectomy, the fibrosis is significantly decreased. Scale bar: 500 µm (**f**) Quantification and comparison of fibrosis between eight weeks and six months after chemical pancreatectomy based on Masson’s Trichrome staining (unpaired t-test *P = .0475).

H&E evaluation of the saline-treated NHPs after surgery showed normal exocrine and endocrine pancreas with normal neurovasculature and ducts (Fig. 2a,b). However, histology after chemical pancreatectomy demonstrated fatty replacement of the pancreas, minimal residual exocrine pancreas, intact neurovasculature, and intact islets of Langerhans (Fig. 2a,b). The saline-treated NHP pancreas demonstrated normal IHC of amylase, insulin, and glucagon throughout the head, body, and tail (Fig. 2c). After chemical pancreatectomy, immunostaining showed a small amount of amylase staining was present in the pancreatic head, but it was otherwise absent in the body and tail. Normal expression of insulin and glucagon was found throughout the pancreas (Fig. 2c), consistent with ablation of the exocrine pancreas and preservation of the endocrine pancreas. Histology-based quantification of the residual exocrine pancreas demonstrated ablation of approximately 85% of the exocrine pancreas (Fig. 2d). In addition, Masson’s Trichrome staining and histological evaluation revealed less fibrosis in the AcA-treated NHPs at six months than the AcA-treated NHPs at eight weeks (Fig. 2e,f).

### EM imaging of the pancreas after chemical pancreatectomy in NHPs

**Figure 3:**
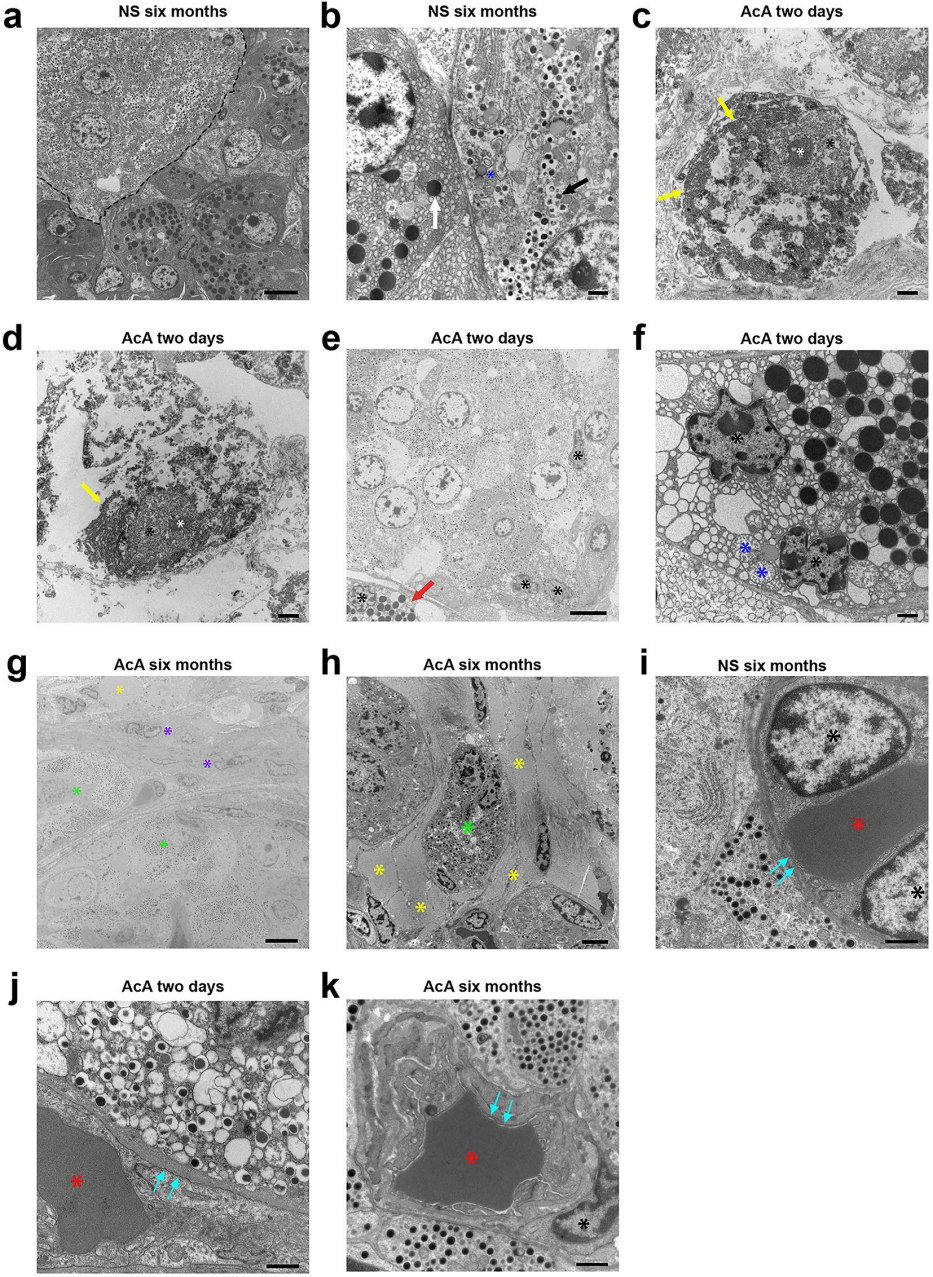
TEM evaluation of the pancreas after AcA or NS pancreatic intraductal infusion in non-human primates. (**a**) Saline-treated NHP pancreas demonstrates normal NHP pancreatic ultrastructure with normal exocrine and endocrine cells. The dashed line indicates endocrine cells. Scale bar: 6 µm (**b**) Higher magnification of saline-treated NHP pancreas demonstrates the typical appearance of normal pancreatic structures: zymogen granules (white arrow), beta granules (black arrow), and normal mitochondria (blue asterisk). (**c** and **d**) Chemical pancreatectomy results in extensive cell death of the head and proximal body of the exocrine pancreas within two days of surgery, with noted degenerative changes of the cytoplasm with areas of clearing, disruptions of the rough endoplasmic reticulum (yellow arrows), and abnormal changes of the nucleus (black asterisk) and nucleolus (white asterisk). (**e**) Chemical pancreatectomy after two days also results in cell injury of the distal body and tail of the exocrine and peripheral endocrine pancreas as denoted by dysmorphic nuclei (black asterisks) and vacuolization of the exocrine pancreas (red arrow). Scale bar: 6 µm. (**f**) Chemical pancreatectomy after two days demonstrates additional signs of cell injury of the exocrine pancreas after AcA, including swollen mitochondria with fragmented cristae (blue asterisks) and dysmorphic nuclei (black asterisk). (**g**) After six months, endocrine cells (green asterisks) are immediately surrounded by fibroblast cells (purple asterisks) and then by adipocytes (yellow asterisk). Scale bar: 6 µm. (**h**) A slightly higher magnification image demonstrates intact endocrine cells (green asterisk) surrounded by adipocytes (yellow asterisks). Scale bar: 4 µm. (**i**, **j**, and **k**) Comparing AcA-treated to saline-treated, TEM demonstrates an intact capillary-endocrine cell interface with intact fenestrations (teal arrows), normal endothelial cell nuclei (black asterisk), and a RBC within the lumen (red asterisk). Scale bars: 1 µm, unless otherwise specified.

EM evaluation of the AcA-treated NHP pancreas correlates with our IHC data. The saline-treated NHP pancreas showed normal ultrastructural features with normal-appearing exocrine pancreas containing zymogen granules, beta-cells containing beta granules, and normal mitochondria (Fig. 3a,b). Two days after the chemical pancreatectomy, there was extensive damage to the exocrine pancreas consistent with cell injury and cell death, including enlargement and disruption of the endoplasmic reticulum, karyorrhexis and karyolysis (Fig. 3c,d), vacuolization of the exocrine cells (Fig. 3e), mitochondrial swelling (Fig. 3f), and changes in nuclear morphology (Fig. 3f). Additionally, two days after AcA, the pancreas body demonstrated an absence of zymogen granules, while Islets of Langerhans appeared normal throughout the pancreas except for the periphery of some islets with dysmorphic nuclei (Fig. 3e). Six months after the chemical pancreatectomy, there were neither zymogen granules nor any evidence of tissue of exocrine origin in the body and tail, and there was extensive fatty replacement with prevalent adipocytes surrounding endocrine cells (Fig. 3g,h).

Lastly, we evaluated the endothelial cells within the islets of Langerhans. The dense fenestrations characteristic of islet endothelial cells (thought to facilitate the exchange of glucose and hormones between the blood and islet endocrine cells) were intact when comparing the saline-treated and AcA-treated NHPs (Fig. 3i–k).

### Chemical pancreatectomy does not alter pain, induce persistent nerve injury, or cause persistent pancreatitis in NHPs

**Figure 4:**
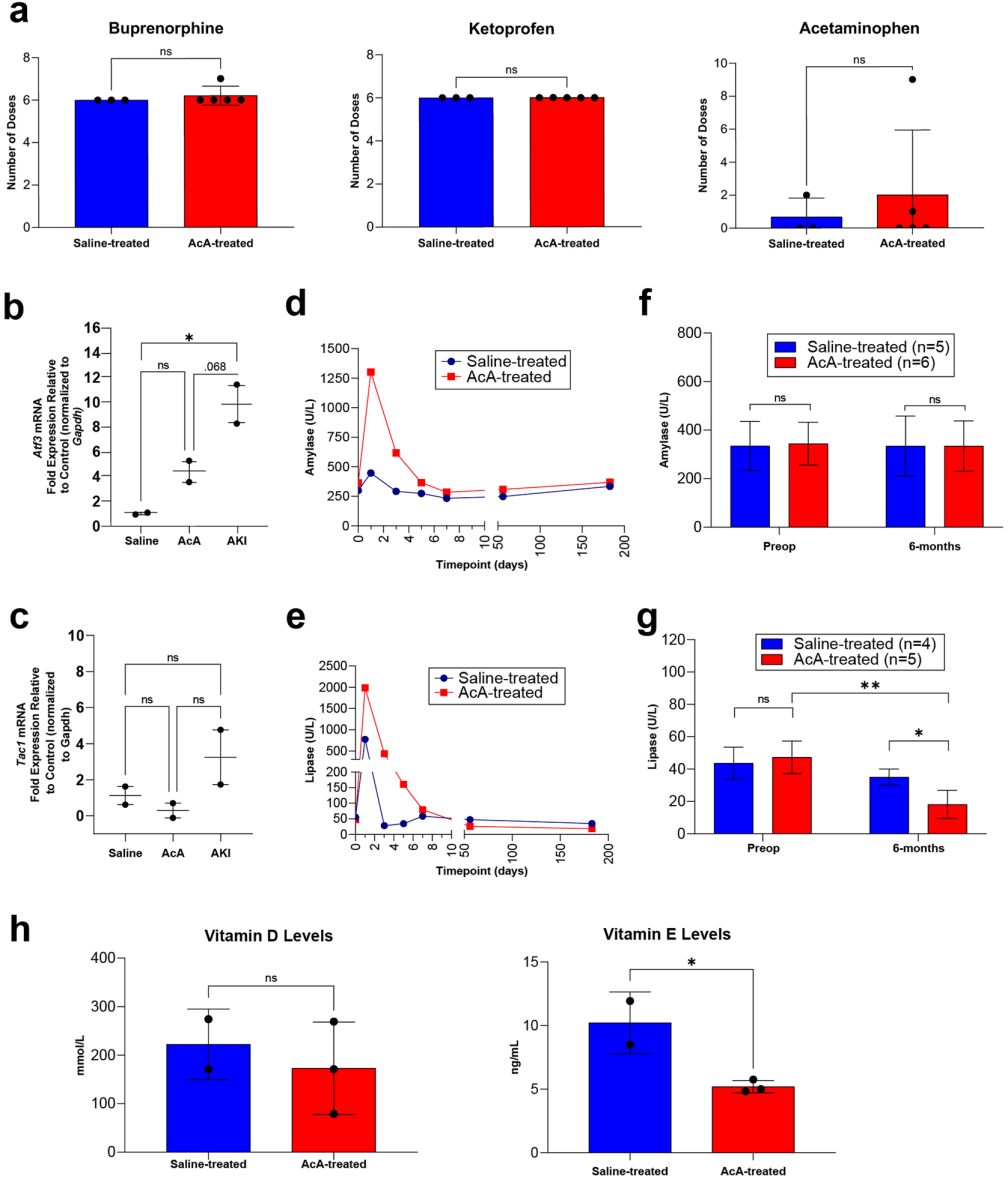

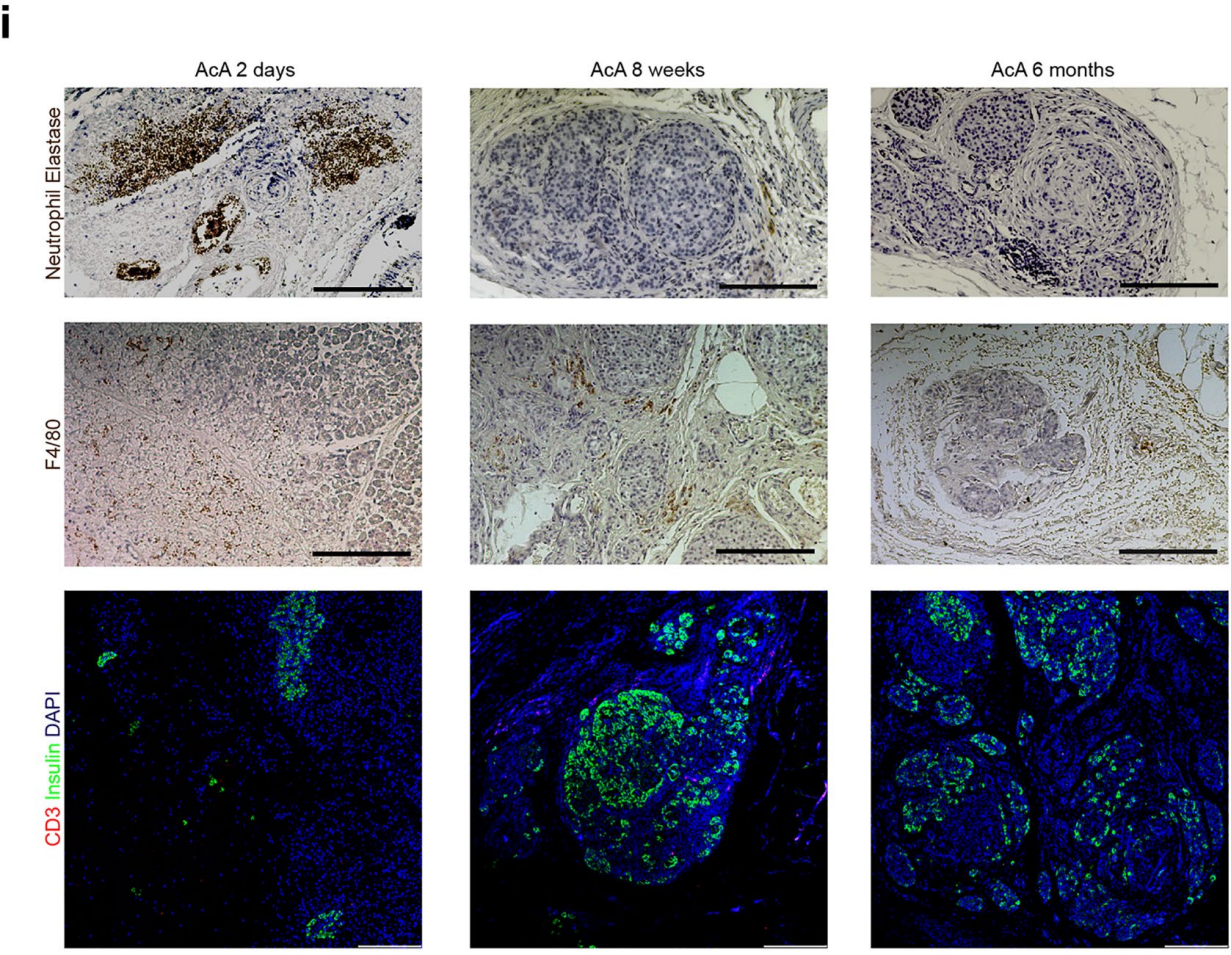
Chemical pancreatectomy does not generate ongoing pain, induce persistent nerve injury, or cause persistent pancreatitis in NHPs. (**a**) There was no significant difference between the number of required doses of buprenorphine (opioid), ketoprofen (NSAID), or acetaminophen after surgery in the saline-treated NHPs compared to the AcA-treated NHPs. (**b**) *Atf3* mRNA is increased in NHPs with AKI compared to saline-treated NHPs (*P = .0186), and there was a trend towards significance when comparing NHPs with AKI to AcA-treated NHPs (*P* = .068). However, there was no difference between the saline-treated and AcA-treated NHPs (*P* = .1949). (Repeated Measure ANOVA with Tukey’s multiple comparisons test). (**c**) *Tac1* mRNA demonstrated no statistical difference between the three groups. However, the mean for the AKI NHPs was higher than the other two groups (repeated Measure One-way ANOVA *P* = .2246). (**d** and **e**) There is normalization of serum amylase and lipase levels one week after the AcA infusion. (**f**) There was no difference between the saline-treated and the AcA-treated NHPs in serum amylase levels at baseline or six months after infusion (unpaired t-test *P* = .4602 and *P* = .9970, respectively). (**g**) Six months after AcA infusion, lipase levels were significantly lower than their corresponding baseline levels (paired t-test **P = .0027) and those of saline-treated NHPs (unpaired t-test *P = .0119). (**h**) Serum vitamin D levels were lower in AcA-treated NHPs than saline-treated NHPs but were not statistically significant (unpaired t-test *P* = .5824). Serum vitamin E levels were lower in the AcA-treated NHPs than the saline-treated NHPs with a statistically significant difference (unpaired t-test *P = .0325) (**I**) Inflammatory infiltrate of neutrophils, lymphocytes, and macrophages is significantly reduced by six months after AcA. Scale bar: 200 µm.

Based on observation and serial evaluations with the onsite veterinarian, the NHP pain behavioral assessment demonstrated no difference in the saline-treated NHPs compared to the AcA-treated NHPs. NHP pain assessment included evaluating NHP activity, food consumption, guarding of the surgical site, posture, changes in respiration, facial expressions, restlessness, and vocalizations^19,20^. For one to two weeks following surgery, all NHPs appeared to have discomfort associated with surgical pain. However, all NHPs required the same amount of pain medications (a combination of buprenorphine (opioid), ketoprofen (NSAID), and acetaminophen). Moreover, the AcA-treated NHPs did not require more pain medication when compared to the saline-treated NHPs (Fig. 4a). To further evaluate markers of nerve injury (*Atf3*) and neurogenic inflammation (*Tac1*), dorsal root ganglia (DRG) were harvested from saline-treated and AcA-treated NHPs six months after surgery. There was no statistical difference in the *Atf3* or *Tac1* mRNA levels when comparing the saline-treated and AcA-treated NHPs. In collaboration with another lab that studies acute kidney injury (AKI), we studied the effects of a laparotomy and nephrectomy on nerve injury and neurogenic inflammation. A nephrectomy results in the axotomy of afferent neurons located in the same dorsal root ganglia as those that innervate the pancreas. Thus, the AKI NHPs served as a positive control. When comparing the saline-treated and AcA-treated NHPs to the AKI NHPs, the AKI NHPs demonstrated higher levels of *Atf3* and *Tac1* mRNA, confirming the presence of nerve injury (Fig. 4b,c) and indicating the absence of nociception and nerve injury in the saline-treated and AcA-treated NHPs.

Bloodwork obtained from the AcA NHPs revealed an elevation of the white blood cell count (WBC) and an elevation of the serum amylase and lipase levels one day following surgery. However, pancreatic enzymes and the WBC count normalized within one week, indicating resolution of the pancreatic inflammation, and remained normal up to six months after surgery (Fig. 4d,e, Supplementary Table 2). Significantly, lipase levels eight weeks and six months after chemical pancreatectomy were statistically lower compared to preoperative levels; this is presumably due to the acinar cells being the primary source of serum lipase. Lipase was also lower than those of corresponding saline-treated NHPs at eight weeks and six months; however, amylase levels did not differ between AcA-treated and saline-treated NHPs (Fig. 4f,g). As expected, consistent with a good ablation of the exocrine pancreas, the NHPs developed some degree of pancreatic insufficiency. Upon evaluating serum levels of vitamin D and vitamin E (Fig. 4h), there were significantly lower levels of vitamin E in the AcA-treated NHPs compared to the saline-treated NHPs. Similarly, the average vitamin D level in AcA-treated NHPs was lower, but this was not statistically different from the saline-treated NHPs. These data may suggest a lack of absorption of lipids.

Consistent with the biochemical changes, two days after AcA treatment, there was an increased inflammatory infiltrate consisting of neutrophils and macrophages (Fig. 4i). Over time, this inflammation was markedly reduced, and there was no evidence of chronic inflammation, as denoted by a lack of T cells assessed via CD3 staining (Fig. 4i).

### Chemical pancreatectomy improves glucose homeostasis in NHPs

**Figure 5:**
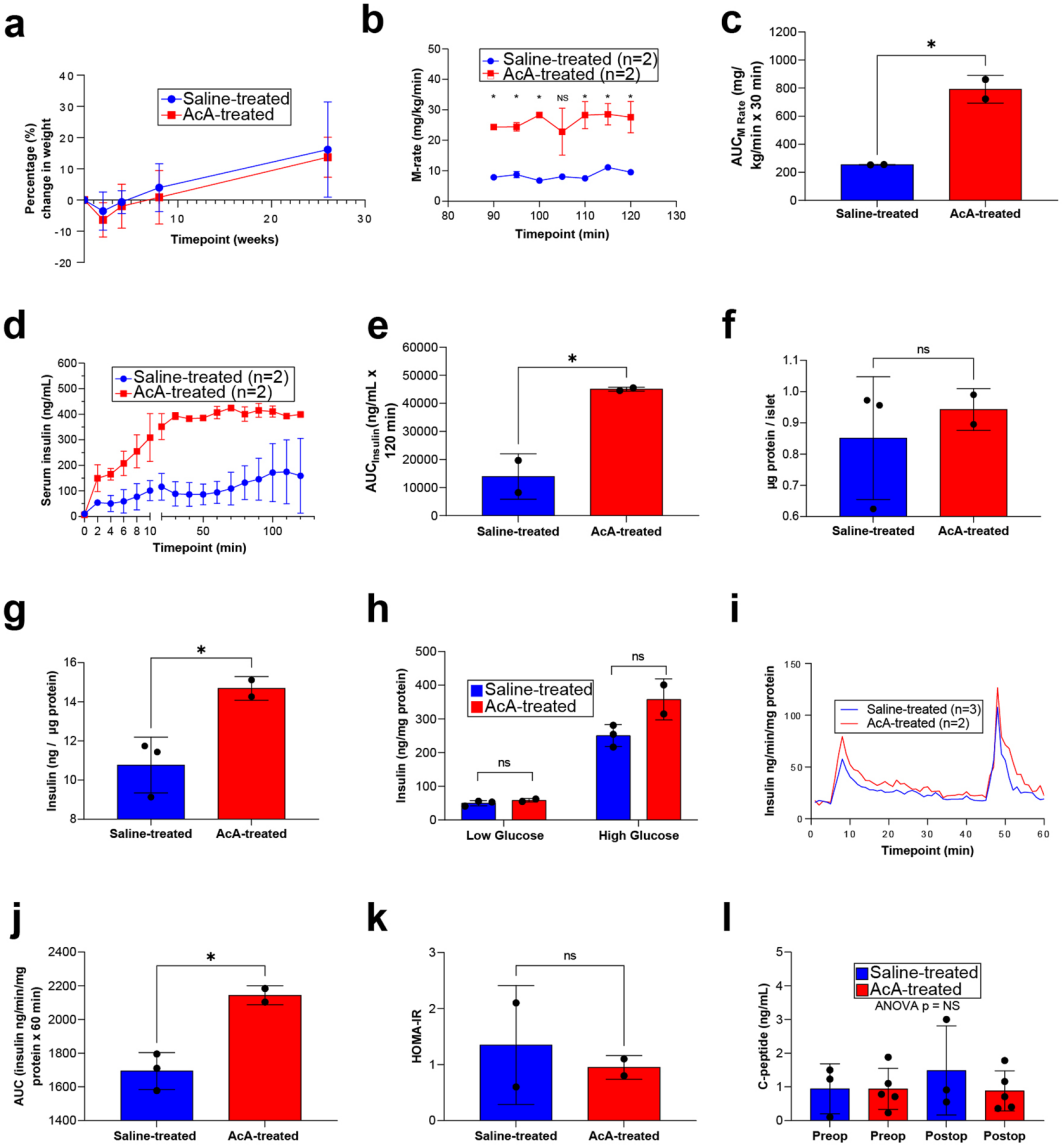
Chemical pancreatectomy improves glucose homeostasis in non-human primates. (**a**) There was no significant difference in weight loss between saline-treated NHPs and AcA-treated NHPs after two weeks. Both groups regain their weight back to baseline by four weeks. Thereafter, the NHPs continue to gain weight above their baseline with no significant difference between the two groups. (**b**) Hyperglycemic clamp in male NHPs demonstrated statistically significant increases in the m-rate (2-way ANOVA *P* = .015) and (**c**) AUC in AcA-treated NHPs compared to saline-treated NHPs (unpaired t-test *P = .0163). (**d** and **e**) Corresponding insulin levels to the hyperglycemic clamp demonstrated a similar increase in AcA-treated compared to saline-treated NHPs (2-way ANOVA *P = .0354). When comparing AUC for insulin levels during the hyperglycemic clamp, there was a significant increase in the AcA-treated NHPs compared to saline-treated NHPs (unpaired t-test *P = .0322). (**f**) There was no difference in islet weight harvested from AcA-treated NHPs compared to saline-treated NHPs (unpaired t-test *P* = .5860). (**g**) Islet insulin content was significantly increased in AcA-treated NHPs compared to saline-treated NHPs (unpaired t-test *P = .0387). (**h**) In vitro* GSIS* showed that islets from AcA-treated NHPs had a trend towards higher insulin secretion compared to saline-treated NHPs (unpaired t-test for low glucose *P* = .2658, for high glucose *P* = .0759). (**i**) Perifusion studies demonstrated increased insulin secretion of isolated islets from AcA-treated NHPs compared to saline-treated NHPs (2-way ANOVA *P* = .0145). (**j**) Perifusion AUC analysis (unpaired t-test *P = .0141). (**k**) HOMA-IR showed no difference in insulin sensitivity between AcA-treated and saline-treated NHPs (unpaired t-t-test *P* = .6532). (**l**) Serum c-peptide levels demonstrated no difference between saline-treated and AcA-treated NHPs preoperatively or postoperatively (2-way ANOVA *P* = .5031).

By the time of sacrifice, all NHPs had gained weight (Fig. 5a). We have previously shown an improvement in glucose tolerance following chemical pancreatectomy in rodents and NHPs^18^. To investigate the cause of the improved glucose tolerance in NHPs, we performed a hyperglycemic clamp to assess the dynamics of insulin secretion. During this study, we monitored the m-rate (“m” for metabolism), which represents the amount of glucose (mg) infused per NHP body weight (kg) per time (min). Thus, m-rate is an indirect measure of insulin levels/secretion. The hyperglycemic clamp six months after surgery revealed that the m-rate was significantly higher in AcA-treated NHPs than in saline-treated NHPs, indicating increased insulin secretion (Fig. 5b,c). Additionally, the actual serum insulin levels measured during the hyperglycemic clamp were significantly higher in the chemical pancreatectomy group compared to saline-treated NHPs (Fig. 5d,e). Next, islets were isolated from NHPs six months post-surgery for in vitro glucose-stimulated insulin secretion (GSIS) and perifusion studies. There was no difference in the islet weight between the two groups (Fig. 5f). We found that the islets isolated from the AcA-treated group had increased insulin content compared to those from the saline-treated (Fig. 5g). In vitro*,* the GSIS assay did not reveal a statistical difference between the saline-treated and AcA-treated islets, but there was a statistical trend (*P* = .0759) upon exposing islets to high-glucose during the GSIS (Fig. 5h). Here, a statistical significance was likely not found because of the small number of animals. The islet perifusion study, a more comprehensive study that illustrates *ex-vivo* dynamic insulin secretion, demonstrated increased insulin secretion in the chemical pancreatectomy islets compared to saline-treated islets (Fig. 5i,j). Next, we calculated HOMA-IR as a marker for insulin sensitivity (Fig. 5k). There was no difference in insulin sensitivity between the AcA-treated NHPs compared to saline-treated NHPs, indicating that the improved glucose tolerance in the AcA-treated NHPs is mainly due to improved beta-cell function. Lastly, we evaluated fasting serum c-peptide levels in saline-treated NHPs and AcA-treated NHPs preoperatively and postoperatively. We found no difference between any of the groups (Fig. 5l).

### Feasibility of chemical pancreatectomy via ERCP in cadavers

To assess the feasibility of this approach in humans, we performed intrapancreatic AcA infusion via ERCP in human cadavers. First, the common channel was cannulated, followed by selective cannulation of the pancreatic duct under fluoroscopic guidance (Supplementary Fig. 3A–C, Supplemental Videos 5–7). Upon verification of correct positioning, we infused a solution of methylene blue and 2% AcA, with a volume of 1.2 cc/mL of pancreatic volume (estimating the average pancreas is 70 mL). We immediately harvested the pancreas and found it uniformly blue and edematous, consistent with perfusion of the entire pancreas (Supplementary Fig. 3D). Importantly, we found that there was no leakage from the minor duct into the duodenum.

## Discussion

Chronic pancreatitis plagues many individuals, and it remains challenging to treat. Current therapeutic options are limited and often sacrifice endocrine function for pain control^21^. Herein, we explored using a chemical pancreatectomy as a potential new therapy to ablate the exocrine pancreas, preserve the endocrine pancreas, and improve glucose homeostasis.

In this study, we found that chemical pancreatectomy occludes the pancreatic ductal system (Fig. 1c). This likely reflects distal ablation of the pancreatic duct. The proximal pancreatic duct may remain patent because of persistent drainage from the proximal, unablated pancreas, or because of the larger caliber of the proximal pancreatic duct. Ablation of the distal pancreatic duct could explain the failure of the exocrine pancreas to regenerate.

Grossly and histologically, we see that the degree of fibrosis is significantly decreased by six months after chemical pancreatectomy compared to eight weeks. Interestingly, we previously saw no significant fibrosis in the mice^18^. This discrepancy likely reflects the difference in the pancreatic architecture and consistency, with the primate pancreas normally containing a more collagenous interstitium, rendering it firmer than the mouse pancreas. Over time, however, we see a significant reduction in the fibrosis, with the six-month time-point showing a similar degree of ablation and minimal residual fibrosis in the NHP pancreas as we previously saw with the mouse pancreas.

One day after the pancreatic intraductal infusion, we observed a rise in the white blood cell count, amylase, and lipase with subsequent normalization within one week. Lipase is a more specific marker for pancreatic injury than amylase because several organs produce amylase (the pancreas, salivary glands, fallopian tubes, gonads, skeletal muscles, adipose tissue, and the intestinal tract)^22,23^. McGeachin et al. demonstrated that multiple species have measurable quantities of amylase within the lungs, spleen, heart, kidneys, muscle, liver, duodenum, and colon. Furthermore, they found that salivarectomized-pancreatectomized rats continued to produce measurable amounts of amylase within the duodenum, colon, kidney, liver, spleen, and lung^24^. Thus, a decrease in the lipase, instead of amylase, below baseline in the AcA-treated NHPs could be used as a marker to assess the degree of exocrine pancreas ablation. Additionally, in view of our data, postoperative serial pancreatic imaging with CT or MRI could also be used as a tool to evaluate the ablation of the exocrine pancreas after chemical pancreatectomy.

Given that the primary purpose of the chemical pancreatectomy is to one day alleviate pain in patients with chronic pancreatitis, we confirmed that the AcA-treated NHPs returned to their baseline behavior and were not different from saline-treated NHPs. In brief, we showed that an AcA infusion did not cause or exacerbate pain. Additionally, the NHPs maintained a normal appetite and continued to gain weight after surgery. Importantly, we are hopeful that this therapy will translate well to those with pain secondary to chronic pancreatitis and prevent the development of widespread central sensitization, which affects 21–28% of patients^25,26^.

We envision that chemical pancreatectomy may not only prevent chronic pancreatitis-related diabetes (CPRD) in chronic pancreatitis patients but may even reverse it. The pathophysiology of CPRD is thought to be related to the toxic milieu of the pancreas combined with extensive fibrosis, which then damages islets and inhibits their function^27^. Previously, we have found improved glucose homeostasis in mice and NHPs following a chemical pancreatectomy^18^**.** To further study the improved glucose homeostasis, we employed the hyperglycemic clamp, the gold standard for evaluating insulin secretion^28^, which showed a significant improvement in insulin secretion in AcA-treated NHPs compared to saline-treated NHPs. We saw this same pattern of improved islet function six months after surgery in AcA-treated NHP islets in vitro using GSIS and islet perifusion. Thus, the improvement in glucose homeostasis associated with the loss of the exocrine pancreas is possibly secondary to the loss of an inhibitory factor secreted by the exocrine pancreas, which functions in a paracrine manner. This substance may normally limit endocrine function, and once the exocrine tissue is removed may then allow the islets to have enhanced function. Given the longstanding improvement in glucose homeostasis observed in mice (8 weeks was the latest time-point evaluated) and NHPs (six months was the latest time-point evaluated), chemical pancreatectomy may improve glucose tolerance in patients with CPRD.

In NHPs, chemical pancreatectomy was associated with few complications. One complication we saw was duodenal narrowing within four weeks of surgery. In NHPs, the head of the pancreas is typically in contact with 50–60% of the circumference of the duodenum, significantly more than in humans. Once this portion of the pancreas becomes fibrotic and edematous, there can be a significant narrowing of the duodenum. This problem is less likely to occur in humans because the human pancreas is in contact with a smaller arc around the duodenum. Additionally, we found that one of 21 (5%) NHPs developed a distal common bile duct stricture and was managed with IV fluids and prophylactic antibiotics. This complication was likely related to infusing into the common channel, where a portion of our infusion inevitably travels into the common bile duct. In humans, this should not happen because a proceduralist will selectively cannulate the pancreatic duct at the time of ERCP, and we would envision a sphincterotomy at the time of the ERCP.

Postoperatively, all NHPs (saline-treated and AcA-treated) develop elevation of pancreatic enzymes; however, optimal postoperative care, including pain medication, anti-emetics, and IV fluids, has generally facilitated clinical recovery. In humans, intensive care unit level monitoring and treatment would allow expeditious treatment of any periprocedural pancreatitis. Interestingly, the AcA-treated NHPs had a much higher rise of their pancreatic enzymes, likely reflecting a more global uncontrolled release as opposed to a localized release secondary to injury as in the saline-treated NHPs. This likely represents a sudden “fixation” and near immediate death of the majority of the acinar and duct cells.

Another nonspecific surgical complication that we encountered was infection. In three of 21 animals, the CT scans demonstrated an intraabdominal abscess. These NHPs were taken back to the operating room for a repeat laparotomy and abdominal washout. Also, two of 21 NHPs developed central line infections, requiring removal of the central lines. Both sets of NHPs did well after their respective interventions, and both sets of complications seem to be related to the laparotomy and duodenotomy and the less-than-ideal aseptic conditions of the animal surgery.

Ideally, this procedure will be translated into humans and carried out via an ERCP. To evaluate the feasibility of this translation, we performed an ERCP in human cadavers. Importantly, we could infuse a large volume entirely through an ERCP approach without surgical intervention; given the blue and edematous appearance of the pancreas post-ERCP, we believe we successfully perfused the entire pancreas. Of note, ERCP-associated pancreatitis occurs in approximately 7% of patients^29^. Because we are immediately “fixing” the tissue upon infusion, we expect the incidence of clinically significant pancreatitis to be much lower.

Upon translation to humans, patients would undergo preoperative imaging in the form of a secretin-enhanced magnetic resonance cholangiopancreatography (MRCP) and possibly ERCP. MRCP would allow for the calculation of preoperative pancreatic volume and provide an overview of ductal anatomy, which would be essential for identifying areas of strictures and stones. ERCP would allow for intervention in the form of lithotripsy or stent placement, which may be necessary before a chemical pancreatectomy. This procedure may require surgical laparoscopic assistance due to, for example, an inability to selectively cannulate the pancreatic duct, requiring infusion through the common channel. Thus, laparoscopically, we would temporarily clamp the common bile duct. Additionally, we have seen that some infusate permeates through the pancreas into the peritoneal cavity and the retroperitoneum. We have found that this permeated infusate is diluted quickly based on pH analysis (Supplementary Table 3). However, one may consider performing a laparoscopic washout of the infusate. Patients with prior pancreatic surgery or any setting where an ERCP could not be performed would be a relative contraindication, considering the same procedure could be performed through a surgical approach as we have described in mice and NHPs.

Approximately 20–60% of chronic pancreatitis patients develop exocrine pancreatic insufficiency and typically require enzyme replacement in the form of pancrelipase, which would have to be continued after chemical pancreatectomy^30^. Symptoms of exocrine pancreatic insufficiency include steatorrhea, weight loss, nausea, vomiting, and abdominal pain^31^. Consistent with pancreatic insufficiency, we found that the AcA-treated NHPs had lower vitamin D and vitamin E levels compared to saline-treated NHPs. Therefore, those not previously taking pancrelipase would likely require initiation after chemical pancreatectomy and may require additional vitamin supplementation. Furthermore, pancrelipase would attenuate the effects of exocrine pancreatic insufficiency, including fat-soluble vitamin deficiency. Sikkens et al. found that vitamin A, D, E, and K deficiencies are present in those with exocrine insufficiency secondary to pancreatitis, with a prevalence of 3, 53, 10, and 63% of patients, respectively. Additionally, these patients often have a significant decrease in bone mineral density resulting in osteopenia and osteoporosis^32^. Vitamin A deficiency often results in night blindness; Vitamin D deficiency is often associated with osteomalacia; vitamin E deficiency is associated with increased oxidative stress; vitamin K deficiency is associated with bleeding^33^. Our NHPs remained on a regular diet and required neither enzyme supplementation nor placement on an elemental diet. We found that in most of the NHPs that underwent a chemical pancreatectomy, a small amount of normal exocrine pancreas remained in the head of the pancreas. This is likely due to NHP pancreatic ductal anatomy differing from human pancreatic ductal anatomy. NHPs may have small ductal tributaries draining directly into the duodenum separate from the main ductal system such that they were unaffected by the acetic acid infusion. Notably, there was no regeneration of the exocrine pancreas by six months after acetic acid as evidenced by the histology and immunostaining of the pancreas.

A limitation of our study is that we did not perform this therapy in a model of chronic pancreatitis in NHPs. Unfortunately, to the best of our knowledge, there is no published model of chronic pancreatitis in NHPs, forcing us to carry out our experiments in normal NHPs. Additionally, given the small size of the NHPs, we were unable to perform the procedure via an ERCP. However, given our success in human cadavers, we do not expect there to be significant difficulties in translating this procedure to humans.

## Conclusion

Here, we demonstrated the efficacy of chemical pancreatectomy in ablating the exocrine pancreas in NHPs, validating our pilot study in NHPs. Furthermore, we tested the feasibility of translating this procedure to humans via an ERCP. We envision that one day in the near future, clinicians will treat chronic pancreatitis differently, utilizing a chemical pancreatectomy, and these patients may prevent or improve their pancreatogenic diabetes.

## Methods

### Animal manipulation

Cynomolgus macaques from both sexes (4–6 years old, weighing 4.5–7.0 kg) were purchased from Alpha Genesis Inc. NHPs were quarantined for 30 days and allowed to acclimate 1–2-weeks thereafter. NHPs were housed with a 12-h light/12-h dark cycle with lights on from 7 am to 7 pm. Animals had free access to water and a diet of biscuits, forage mix, fruits, and vegetables. Endocrine assays were performed on males.

### Pancreatic ductal infusion in NHPs

Surgeries were performed as previously described^18^. Animals were put in a jacket and tether system for two weeks before surgery to allow the NHP to acclimate to the system. Before surgery, the NHP was sedated using ketamine (10 mg/kg intramuscular), intubated, and administered inhalational anesthesia with isoflurane. Following a tunneled central venous catheter placement into the right internal jugular vein (Supplementary Fig. 1C), we performed a midline laparotomy and placed a temporary clamp on the common bile duct to prevent infusion into the liver and gallbladder. Next, a duodenotomy was performed to allow for the identification of the major papilla. We then cannulated the common pancreaticobiliary channel using an umbilical catheter and secured the catheter using a bulldog clamp, preventing backflow into the duodenum. Subsequently, we infused methylene blue solution, identified any solution leakage, and controlled this with additional bulldog clamps. The contrast was injected through the catheter, and fluoroscopy was used to confirm adequate placement of the catheter and patency of the pancreatic duct. Next, we infused a volume of 1.7 mL/kg of 2% AcA or normal saline (NS) via an umbilical catheter at a rate of 2 mL/min. Acetic acid was then allowed to dwell within the pancreas for 10 min before removing the catheter and clamps and closing the duodenotomy. The laparotomy was then closed, and the jacket and tether system reapplied. NHPs received pain medication, ondansetron, famotidine, and IV fluids postoperatively until adequate oral intake was re-established.

### Acute kidney injury (AKI)

In collaboration with a separate lab that studies AKI in NHPs, using age-matched Cynomolgus macaques, ischemia–reperfusion was performed by clamping the right kidney for approximately one hour while a left nephrectomy was performed. These NHPs were sacrificed after seven days to evaluate the kidney for histology. The lab allowed us to evaluate the pancreata of these NHPs and evaluate them for markers of nerve injury. Additionally, these pancreata were used as controls for in vitro islet studies.

### Hyperglycemic clamp

The hyperglycemic clamp was completed in NHPs as previously described^34,35^. Following overnight fasting, NHPs were sedated (ketamine, 10 mg/kg intramuscular). Two vascular catheters were placed: one for the continuous infusion of the 20% dextrose solution; the second to collect blood at serial time points for serum insulin measurements. Capillary blood glucose was measured from the NHP’s tail tip serially. The capillary blood glucose concentration was raised to 125 mg/dL above baseline and maintained at that level for the duration of this procedure through adjustments of the infusion rate every five minutes. The glucose solution was continuously infused throughout the experiment, with adjustments in the rate calculated every five minutes to maintain the desired glucose level. Serial blood samples were collected for the designated time points for serum insulin measurements.

### NHP islet isolation

The islet isolation protocol was adapted for NHP pancreata^36^. Total pancreaticoduodenectomy was performed following NHP sacrifice. Harvested pancreata were transferred to cold University of Wisconsin (UW) solution with cold ischemia time averaging 20 min. After removing non-pancreatic tissue, the pancreatic tissue was washed in an antibiotic solution and weighed. The pancreatic neck was transected, and catheters were placed proximally and distally. Both catheters were secured with suture. We infused a blend of exogenous enzymes [collagenases and neutral proteases (Vitacyte)] into the pancreas and then transferred them into the Ricordi digestion chamber (Biorep Technologies Inc.) for mechanical disruption by shaking. After digestion, islets and pancreatic cells were washed in a cold RPMI solution supplemented with human serum albumin. The islets were purified using a polysucrose discontinuous gradient, washed, hand-picked, and cultured at 37 °C overnight before performing functional analyses.

### In vitro glucose-stimulated insulin secretion (GSIS)

Isolated islets (AKI NHP n = 2, control NHP n = 1, AcA NHP n = 2) recovered overnight at 37 °C in CMRL-1066 media containing 10% fetal bovine serum and 2 mmol/l L-glutamine. GSIS was performed as previously described^18^. Briefly, groups of 30 islets per NHP were incubated in 2.8 mM glucose for 30 min at 37 °C to establish a stable basal insulin secretion and were then washed with Krebs buffer twice. The islets were transferred into a new well containing 2 ml of 2.8 mM glucose solution for 30 min at 37 °C, and 100 μl of media was collected for time point 1. The islets were then transferred into a new well containing 2 ml of 20 mM glucose solution for 30 min at 37 °C, and 100 μl of media was collected for time point 2. The islets were then recovered for protein quantification. Insulin levels in the collected media were measured using the human insulin ELISA kit (ALPCO) and were normalized to the protein content.

### Islet perifusion assay

Isolated islets (from the same groups as above) recovered overnight in CMRL-1066 medium (Gibco) containing 10% fetal bovine serum and 2 mmol/l L-glutamine at 37 °C. Thirty islets per NHP were placed in a dynamic perifusion system (Amersham Biosciences AKTA FPLC System) as previously described^37^. To summarize, the perifusion was performed using Krebs buffer with 2.8 mM glucose at a flow rate of 1 ml/min for 30 min to establish stable basal insulin secretion. Next, the islets were perifused with 2.8 mM glucose for 10 min, and fractions of 500 μl were collected every 30 s. Then, the glucose concentration was increased to 20 mM, and fractions of 500 μl were collected every 30 s for 20 min. Finally, the islets were perifused with 30 mM KCl, and fractions of 500 μl were collected every 30 s for 10 min. After the perifusion, the islets were recollected from the column for protein quantification. The insulin in the effluent was measured as described above. The fractional insulin secretion rate was calculated as secreted insulin per minute normalized to the protein content.

### Measuring insulin content from isolated islets

Thirty equal-sized islets per NHP were incubated in 100 μL acid/ethanol (75% ethanol and 0.15 M HCl) at 4 °C overnight as previously described^37^. Gentle rotation was used to extract insulin, followed by centrifugation at 14,000 rpm for 10 min. The supernatant was diluted to a 1:50 ratio, and the insulin content was measured as above and normalized to the total islet protein content.

### Assessment of pancreatic volume via computed tomography (CT) scan

NHPs were sedated and prepared as before surgery^18^, oral contrast (gastrografin) was administered via an orogastric tube, and IV contrast was administered via an IV catheter. CT scans were performed preoperatively, two weeks, four weeks, eight weeks, and six months after surgery. Two separate investigators measured pancreatic volume by creating a 3-D reconstruction of the pancreas using Vitrea software as described^38^. A radiologist verified these measurements.

### Measurement of serum pancreatic enzymes, comprehensive metabolic profile (CMP) and complete blood count (CBC), vitamin D levels, vitamin E levels

Blood was collected to measure pancreatic enzymes, CMP, and CBC as previously described^18^. Blood was collected from the central line or peripherally from the saphenous vein after the placement of a peripheral angiocatheter. Pancreatic enzymes and comprehensive metabolic profile were measured using the Dimension Vista 500 chemistry analyzer (Siemens Medical Solutions USA Inc.). Serum vitamin D and vitamin E levels were measured by IDEXX BioAnalytics.

### Tissue processing and histology

Pancreatic tissues were processed and evaluated for IHC as described^18^. Pancreas samples were fixed with 4% paraformaldehyde (PFA) for 24 h at 4 °C. The tissues were then embedded into paraffin, and 5 μm sections were cut. For IHC, antigen retrieval was performed as appropriate using heat and citrate buffer as described. Slides were incubated with the following primary antibodies: Guinea pig insulin (Abcam ab195956, 1/500), Rabbit amylase (Sigma a8273, 1/300), Mouse glucagon (Abcam ab10988, 1/1000), Rabbit CD3 (DAKO a045229-2, 1/75), Rabbit neutrophil elastase (Abcam ab68672, 1/200), Rat F4/80 (Abcam ab6640, 1/200) at 4 °C overnight. On the subsequent day, slides were incubated with fluorescent-conjugated (CY2, CY3, CY5) secondary antibodies at a concentration of 1/300 (Jackson ImmunoResearch Labs) for 1 h at room temperature. Mounting and nuclear staining were performed using Fluoroshield with DAPI (Sigma-Aldrich). Alternatively, following incubation with primary antibody, slides were washed and incubated with goat anti-rabbit horseradish peroxidase conjugated secondary antibody (1:1000, Abcam, ab97051) for 1 hour at room temperature, followed by DAB, hematoxylin, and bluing solution. Slides were then dehydrated and mounted using Cytoseal 60. Additionally, five μm sections of paraffin-embedded pancreata were stained with H&E and Masson’s Trichrome stain for histological assessment.

### Transmission electron microscopy

We performed transmission electron microscopy (TEM) as previously described^18^. The tissue is fixed in MJK solution and rinsed three times for fifteen minutes each in a 0.1 M Sodium Cacodylate buffer. The tissue is then placed in a 1% osmium tetroxide for 1 h. The tissue is then rinsed with 50% ethanol, and then sequentially dehydrated in 50% ethanol, 75% ethanol, and 95% ethanol for 15 min each. Lastly, the tissue is placed in 100% EtOH (15 min × 2). Next, the tissue is placed in propylene oxide for (15 min × 2). Then the tissue was preinfiltrated with a 2:1 propylene oxide resin for 1 h, then 1:2 propylene oxide resin for 2 h, and then pure resin for 1 h. Lastly, the tissue is embedded and polymerized at 60 degrees C overnight. The embedded tissues were sectioned with a Leica EM UC6 ultramicrotome at a thickness of 90 nm and collected on copper mesh grids. The following day, the tissue is stained with alcoholic uranyl acetate for five minutes and then Reynolds lead citrate for five minutes. TEM imaging was performed on a Philips EM 208 microscope at 60 kV using a Hamamatsu digital camera.

### Isolation of RNA and qPCR

DRG (T8-T12) were harvested and snap-frozen. RNA was isolated using Trizol reagent (Invitrogen). 0.5 µg RNA was used to synthesize cDNA using iScript cDNA synthesis kit (BioRad). Primers were designed using NCBI and targeted to Macaca fascicularis *Gapdh* (accession no. KM491710), *Atf3* (XM_005540783), and *Tac1* (AB220474). Primers were validated using Monkey Universal Reference cDNA (Zyagen). Gel electrophoresis was used to confirm that PCR products were of the predicted size without dimerization or nonspecific bands. Sybr green PCR amplification (Universal Sybr Green Supermix, Bio-rad) was performed using a Bio-Rad CFX Connect real-time system. After amplification, a dissociation curve was plotted against the melting temperature to ensure the amplification of a single product. All samples were run in duplicate. The negative control was the amplification of a control reaction product (product of reverse transcription of master mix in the absence of template cDNA). The relative fluorescence of SYBR green was compared with a passive reference for each cycle. Cycle time (Ct) values were determined via regression and recorded as a measure of initial template concentration. Relative changes (ΔCt) in mRNA levels were calculated using *Gapdh* as a reference standard. Fold expression was determined using the ΔΔCt method.

### Endoscopic retrograde cholangiopancreatography (ERCP) in cadavers

We inserted a side-viewing endoscope into the oropharynx of the supine cadaver, advanced through the esophagus, and into the stomach. The scope was then passed through the pylorus and into the duodenum. The ampulla was identified and cannulated. The cadaver was then repositioned into the prone position, and fluoroscopy was used to ensure proper position. Next, under fluoroscopic guidance, contrast was used to identify the pancreatic ductal system. We then infused a 20% methylene blue 2% AcA solution into the pancreas. We chose to infuse a volume of 1.2 cc/mL of the pancreas (estimating the average pancreas is 70 mL).

### Graphics

Gross images were taken with a Sony Macro Lens and camera. Figure 1a was created with https://www.biorender.com/. IHC images were captured using Leica STELLARIS 5 confocal system (using the 10 × objective) with Leica Application Suite X (LAS X) at the CHP Rangos imaging core facility. Brightfield imaging was obtained using the Thermo Fisher EVOS M7000 system (using the 10 × and 20 × objectives) with EVOS M7000 Software revision 2.0.2094.0. Whole H&E slides were scanned using the Leica Aperio AT2 system (using the 40 × objective) and subsequently evaluated using Aperio ImageScope 12.4.3. Images were optimized using Adobe Photoshop (version 23.3.1). Figures were compiled using Adobe Illustrator (version 26.2.1).

### Quantification of the exocrine pancreas

NHP pancreas slides were scanned using the EVOS m7000 Software using the 20 × objective. ImageJ was then employed to measure the total pancreatic area and the area of the exocrine pancreas, evaluating the head, body, and tail of saline-treated and AcA-treated NHPs.

### Quantification of fibrosis

Masson's Trichrome stained NHP pancreas slides were scanned using EVOS m7000 Software using the 20 × objective. ImageJ was then utilized to quantify the areas of fibrosis across the body and tail of multiple saline-treated and Aca-treated NHPs.

### Study approval

The Animal Research and Care Committee at the Children’s Hospital of Pittsburgh and the University of Pittsburgh Institutional Animal Care and Use Committee approved all NHP experiments, which were carried out in accordance with their respective guidelines and regulations. All methods are reported in accordance with ARRIVE guidelines. The Humanity Gifts Registry Program provided the cadavers, and all cadaveric experimental protocols were approved by the University of Pittsburgh, and all methods involving the cadavers were carried out in accordance with the University of Pittsburgh guidelines and regulations. Informed consent was obtained from all donors or the next of kin by the Humanity Gifts Registry.

All authors had access to the study data as well as reviewed and approved the final manuscript.

### Calculations and statistics

Homeostatic Model Assessment for Insulin Resistance (HOMA-IR) [(Fasting Glucose x Fasting Insulin)/22.5] was calculated^39^. AUC was calculated by the trapezoidal method. Data are displayed as mean $\pm$ SD. Comparisons between groups were made using unpaired, paired 2-tailed t-tests, or 1- or 2-way ANOVA as indicated, followed by the indicated post hoc test for multiple comparisons. *P* ≤ .05 was considered statistically significant. Statistical tests were conducted using GraphPad Prism, version 9.3.1.

### Ethical approval and consent to participate

The Animal Research and Care Committee at the Children’s Hospital of Pittsburgh and the University of Pittsburgh Institutional Animal Care and Use Committee approved all NHP experiments, which were carried out in accordance with their respective guidelines and regulations. All methods are reported in accordance with ARRIVE guidelines. The Humanity Gifts Registry Program provided the cadavers, and all cadaveric experimental protocols were approved by the University of Pittsburgh, and all methods involving the cadavers were carried out in accordance with the University of Pittsburgh guidelines and regulations. Informed consent was obtained from all donors or the next of kin by the Humanity Gifts Registry.

## Supplementary Information

Supplementary Video 1.

Supplementary Video 2.

Supplementary Video 3.

Supplementary Video 4.

Supplementary Information 1.

## Data Availability

Data, analytic methods, and study materials available upon request. Please contact the corresponding author for additional information.
