# Supplementary material for: Chemical pancreatectomy in non-human primates ablates the acini and ducts and enhances beta-cell function
Source: Sci Rep. 2023 Jun 5;13:9113. doi: 10.1038/s41598-023-35820-2 (PMC10241801; doi:10.1038/s41598-023-35820-2)
Supplement: Supplementary file 5 — Supplementary Information 1. [file 41598_2023_35820_MOESM5_ESM.docx]

Figure S1

#
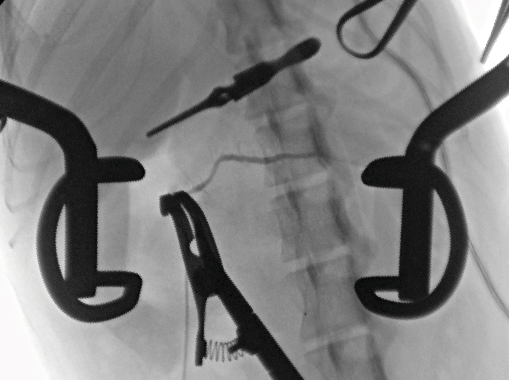

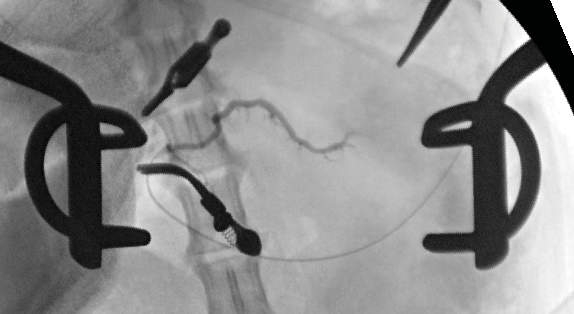
a b


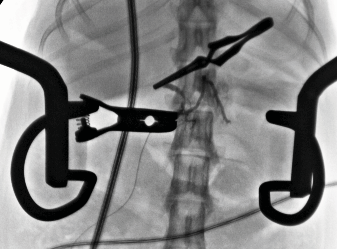

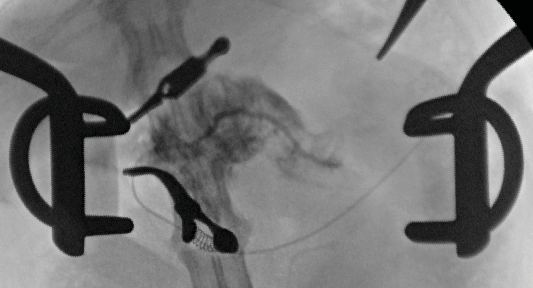


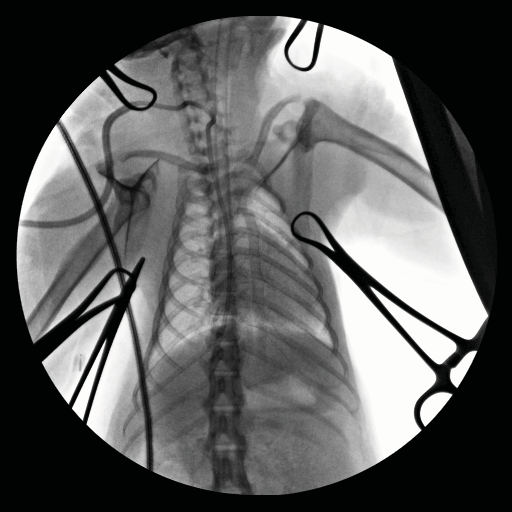
**c**

**Figure S1: Fluoroscopic evaluation during surgery**

**(a)** Pancreatography. **(b)** Pancreatography demonstrating acinarization (before and after). **(c)** Fluoroscopic confirmation of appropriate central venous catheter placement.

Figure S2

# a b c

10


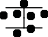

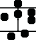


✱✱✱✱

15

10

**Volume (mL)**

5

0

0 10 20 30

**Time After Surgery (weeks)**

# d

8

6

**Volume (mL)**

4

2

0

0 10 20 30

**Time after Surgery (weeks)**

# e

8

6

**Volume (mL)**

4

2

0

**Preop CT 8 weeks postop**

10


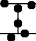


ns

**NHP Body Weight (kg)**

8 20

CT-Based Pancreatic Volume

Actual Pancreatic Volume

6 15

**Volume (mL)**

4 10

2 5

0 0

**Saline-treated AcA-treated**

**Figure S2: Additional CT-based pancreatic volume analyses and comparison to pancreatic weight and pancreatic volume (by displacement)**

**(a, b)** CT-based pancreatic volumes over time for individual saline-treated NHPs **(a)** and AcA-treated NHPs **(b)**. **(c)** CT-based pancreatic volume is significantly lower in the AcA-treated NHPs compared to saline-treated NHPs at eight weeks (paired t-test ****P <.0001). **(d)** There was no significant difference in body weight between the saline-treated and AcA-treated NHPs (unpaired t-test P=.8566). **(e)** Comparison between CT-based pancreatic volumes and actual pancreatic volumes measured by displacement were similar.

Figure S3

# a b c


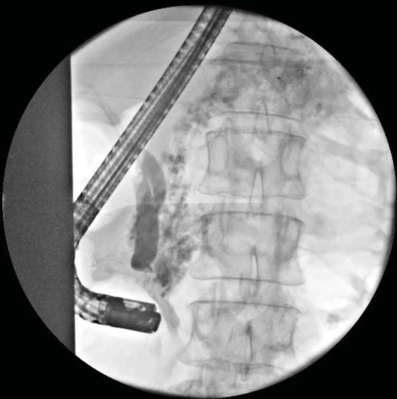

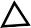

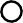


*


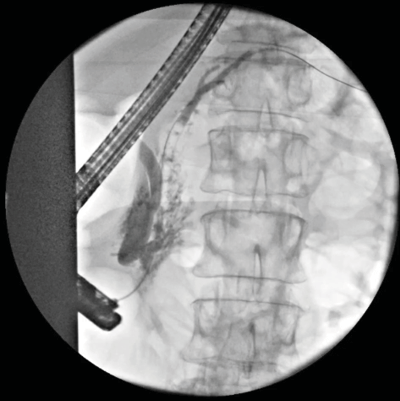

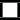

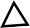

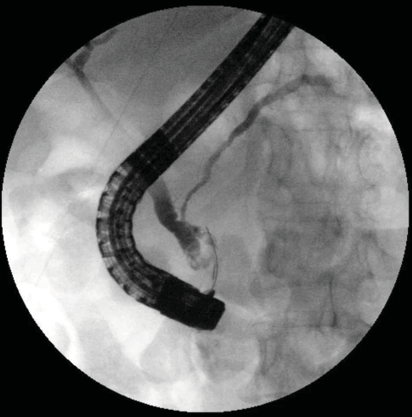

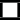

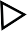


**d**


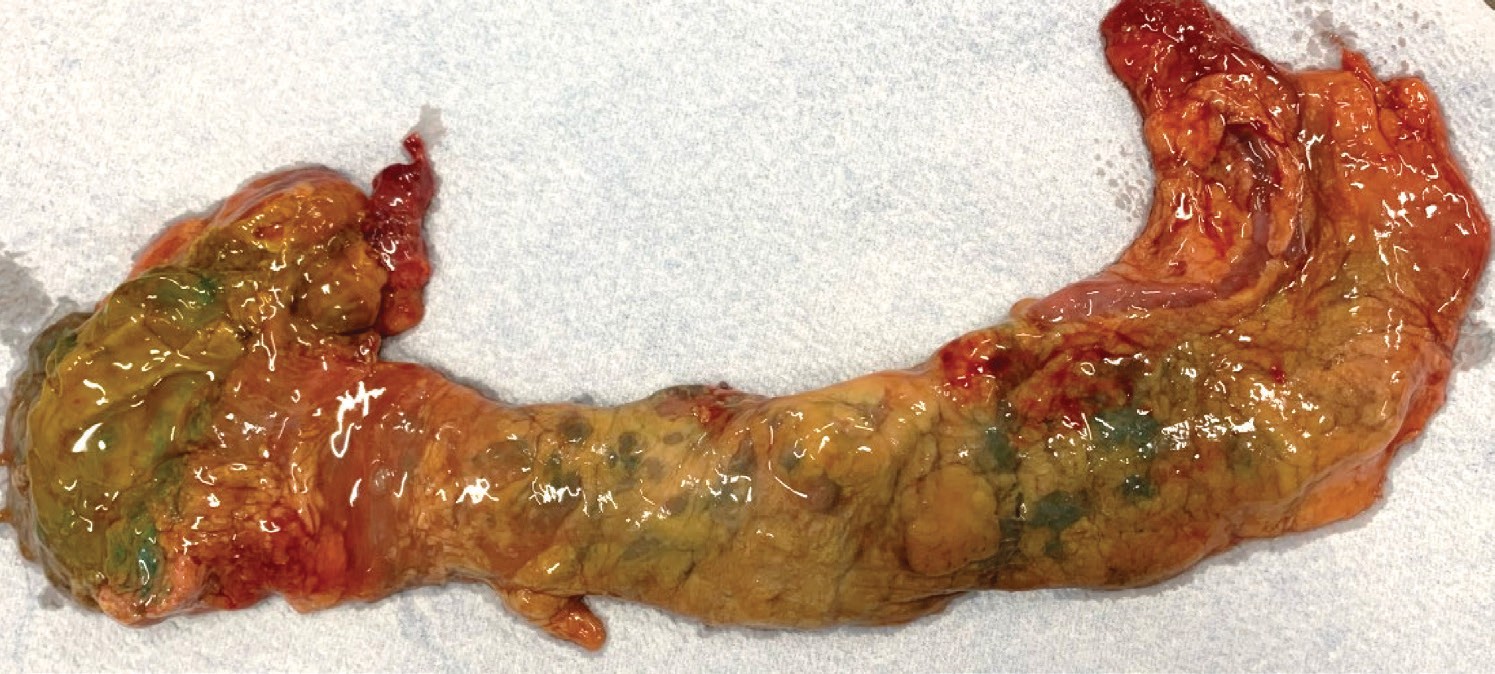


*


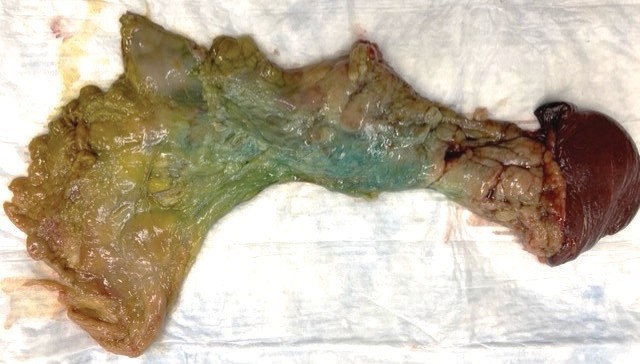


**Figure S3: Feasibility of chemical pancreatectomy via ERCP in cadavers (2% AcA with methylene blue)**

(a) ERCP illustrates the filling of the common bile duct (triangle arrowhead) with a medial take-off of the cystic duct (circle arrowhead) with noted acinarization of the pancreas (asterisk). (b) ERCP showed the filling of the CBD (triangle arrowhead) and the pancreatic duct (square arrowhead) with a wire in place, demonstrating selective cannulation of the pancreatic duct. (c) ERCP demonstrated the filling of the common bile duct and pancreatic duct. (d) The gross cadaveric pancreas was harvested immediately after infusion, and it was grossly blue and edematous from head to tail. The duodenum remained attached (asterisk).

Table S1: Table listing pancreatic weights and volume at time of sacrifice

| **NHP** | **Sex (M/F)** | **Body weight (kg)** | **Pancreas volume (ml)** | **Pancreas weight (g)** | **Pancrea�c Volume (mL) / NHP body weight (kg)** |
| --- | --- | --- | --- | --- | --- |
| M3-20 (AcA) | M | 4.8 | 3.25 | 3.3 | 0.677083 |
| M12-20 (AcA) | M | 6.6 | 6 | 5.875 | 0.909091 |
| M261-20 (AcA) | M | 6.3 | 4.5 | 4.2 | 0.714286 |
| M260-20 (AcA) | M | 6.5 | 1.1 | 1.048 | 0.169231 |
| M257-20 (AcA) | F | 5.4 | 3 | 3.18 | 0.555556 |
| M262-20 (AcA) | F | 4.56 | 3 | 3.11 | 0.657895 |
| M2-20  (Control) | M | 4.5 | 5.30 | 5.27 | 1.177778 |
| M21-20  (Control) | M | 5.9 | 14 | 13.672 | 2.372881 |
| M4-20  (Control) | M | 5.46 | 10.029 | 9.6 | 1.836813 |
| M17-20  (Control) | M | 5.8 | 8.814 | 8.6 | 1.519655 |
| M18-20  (Control) | M | 6.25 | 7.174 | 6.4 | 1.14784 |
| M19-20  (Control) | M | 5.76 | 5.783 | 5.5 | 1.003993 |

Table S2: Tables listing average CMP and CBC before and after chemical pancreatectomy

| **Paired t**  **Six months test p- Pre-op (±SD) post-op (±SD) value** | | | |
| --- | --- | --- | --- |
| **Na** | 144±1.41 | 143.67±0.94 | 0.84 |
| **K** | 4.77±0.26 | 5±0.16 | 0.37 |
| **Cl** | 112.67±2.62 | 113±2.16 | 0.67 |
| **Ca** | 8.47±0.42 | 9.23±0.09 | 0.14 |
| **Lipase** | 49±9.09 | 16.33±8.38 | 0.02 |
| **Glucose** | 45.67±8.34 | 50.33±4.03 | 0.47 |
| **Amylase** | 424.67±48.94 | 416±29.39 | 0.74 |
| **CO2** | 23.33±2.05 | 23±0.82 | 0.87 |
| **Albumin** | 3.03±0.09 | 3.43±0.12 | 0.02 |
| **BUN** | 14.67±1.89 | 16.67±1.89 | 1.48E-16 |
| **Creatinine** | 0.68±0.22 | 0.74±0.17 | 0.28 |
| **ALT** | 40±4.55 | 47±12.33 | 0.41 |
| **AST** | 44.67±9.81 | 49±11.58 | 0.74 |
| **TP** | 6.30±0.14 | 6.97±0.31 | 0.08 |
| **Tbil** | 0.23±0.05 | 0.33±0.12 | 0.42 |
| **Alk phos** | 693±148.6 | 880.33±128.25 | 0.29 |
| **Prealbumin** | 13.67±1.35 | 12.73±1.07 | 0.61 |

| **Pre-op (±SD)** | | **Six months post-op (±SD)** | **Paired t test p- value** |
| --- | --- | --- | --- |
| **WBC** | 7.47±1.28 | 10.8±3.69 | 0.41 |
| **Hemoglobin** | 11.97±1.72 | 12.93±1.22 | 0.13 |
| **Platelets** | 411.67±92.87 | 343±17.8 | 0.33 |

Table S3: Changes in infusate in peritoneum over time

|  | |
| --- | --- |
| pH of 2% AcA: | 2.2 |
| pH of peritoneal ﬂuid after 3 minutes: | 4.78 |
| pH of peritoneal ﬂuid after 8 minutes: | 6.77 |
